# Training processes of World Masters Orienteering Championship medalists

**DOI:** 10.1371/journal.pone.0333126

**Published:** 2025-09-25

**Authors:** Piotr Cych, Weronika Machowska-Krupa

**Affiliations:** Department of Physical Education and Sport, Wroclaw University of Health and Sport Sciences, Wroclaw, Lower Silesia, Poland; ISSEP Kef: Universite de Jendouba Institut Superieur du Sport et de l'Education Physique du Kef, TUNISIA

## Abstract

The study aimed to capture the relationships between sports performance and workouts in different foot orienteer age groups. The preparation process of 49 World Masters Orienteering Championship (WMOC) medalists (aged 35–75) was surveyed using a questionnaire. The respondents were asked about the number of training sessions, time devoted to training and competitions, and the characteristics of the training measures used (including the number of specialized training sessions and competitions) during the preparation and competition periods. The data underwent non-parametric statistical analysis, with the Mann-Whitney U test used to assess differences between two structural indicators. Men trained more frequently than women (*p = *0.015), and younger subgroups (WM35-WM55) trained more often than older ones (WM60-WM75) (**p* *= 0.021). During preparation, men trained longer than women (**p* *= 0.045), while younger masters trained more hours than older ones. No significant gender differences were found in training volume during the competition period (**p* *= 0.74). Sixty-two percent of medalists used over 10 specialized training sessions within six months of WMOC, with younger male veterans (M35-M55) reporting the highest number. More men (92.9%) than women (54.5%) competed over 10 times in the six months preceding WMOC (**p* *= 0.002). WMOC medalists trained optimally, considering age-related decline, and frequently prepared by participating in orienteering competitions. Some training work-out differences were observed among medalists based on age and sex. The study observations provide the first insight into the preparation of master category orienteering competitors for international competition. Indeed, most are very professional in their preparation and devote much time to training and participating in competitions, especially younger competitors.

## Introduction

Sports for older adults have become more popular globally in recent decades, especially in highly developed countries [1,2]. The issue of aging and its impact on the ability to compete has been, and remains, the subject of numerous studies and scientific works [3–5], with master athletes practicing endurance sports repeatedly observed [6–10]. However, each sport has its specificity, which is best understood by characterizing current or former champions. After all, they were, and often still are, the best at what they do. This is the case with orienteering, which involves finding control points in the terrain as quickly as possible in a given order based on the content of a specialized map while engaging cognitive processes [11,12]. Orienteering is an endurance sport in which several factors influence the final result, including endurance, running speed, the ability to quickly and accurately choose a variant based on the information contained in the map, and finding control checkpoints [13–16].

Examining the world’s best competitors, all of whom gather in one place every year during the World Masters Orienteering Championships (WMOC), provides a deeper understanding of the sport and the methods required to achieve success. The WMOC attracts several thousand participants annually and has been held almost every year for 40 years, with the first competition taking place in Finland in 1983. There are so many competitors because orienteering meets the needs and capabilities of older adults [17,18]. WMOC participants are usually divided into 25 classes, with the oldest being 96 years old. Participation is open to everyone aged 35 years or older and is not usually restricted in any other way (representatives of Belarus and Russia were not allowed to participate in the WMOC). Participants compete in classes based on five-year age intervals (e.g., the M35 class is for men competitors aged 35–39). An additional group stratification criterion is sex, with men and women competing separately. Three individual competitions take place during the championships over three distances (sprint, middle, and classic) and are preceded by qualifications. Holding three final heats (sprint, middle, and long) makes it possible to select more than three medalists in one class (a maximum of nine – assuming that none are repeated and there is no more than one competitor in third place), resulting in over 200 medalists across 25 classes, though many medalists win over more than one distance.

Sports science assumes a significant relationship between training load and results [19]. Therefore, sports training should be organized in such a way that the highest goals can be achieved at the lowest possible cost (e.g., energy and time). It can be assumed that the best athletes in the world operate according to this rule, and their sports training can serve as a universal model of the relationship between input and achieved effects. An excellent example of such behavior is modeling the training carried out by the world’s best marathoners, all of whom train for several hundred hours and run several thousand kilometers a year, both in the year of their greatest achievements and the preceding years [20–22]. Therefore, it is important to understand master orienteers’ training practice, including training volume and characteristics (e.g., specialist map training and competition participation frequency).

The study aimed to capture the relationships between sports performance and workouts in different age groups of master foot orienteers. Since there is no information in the present literature on the preparation process among master orienteers, the current research aimed to determine the structure (training volume, intensity, and specificity) of the WMOC preparation process and compare it to elite orienteering training. For this purpose, analyses were carried out to answer the following questions:

What was the average weekly training frequency of WMOC medalists?

Was training frequency related to the age and sex of the medalists?

How many hours per week did they typically devote to training?

How often did WMOC medalists use specific training to prepare for the WMOC?

How often did WMOC medalists participate in orienteering competitions before WMOC?

## Materials and methods

### Participants

The study participants were medalists at the WMOC, an inclusion condition that ensured a high level of sportsmanship among the respondents. The WMOC is open to anyone who wants to and can afford to participate, resulting in several thousand participants each year competing across a range of age classes that can contain over 200 athletes. Such a large number of participants in individual classes means that only the best will take a place on the podium. The experience of competitors varies, but the medalists typically started orienteering training in their youth, with a median age of 15 years for men and women (Table 1). However, the standard deviation is high due to individuals commencing training at 60 years old, especially women.

**Table 1:** Somatotypes and sports careers of WMOC medalists.

Sex	Body height [m]	Body mass [kg]	BMI [kg/m^2^]	Initial competing age [years]	Initial orienteering training age [years]
**Men** **(n = 28)**					
Mean	1.79	68.86	21.51	16.50	17.40
Median	1.80	70.00	21.34	13.00	15.00
SD	0.05	04.93	01.43	10.18	08.95
Min.	1.67	58.00	18.70	07.00	10.00
Max.	1.88	78.00	24.07	50.00	55.00
**Women** **(n = 21)**					(n = 20*)
Mean	1.65	56.00	20.43	21.95	23.55
Median	1.65	54.00	20.03	16.00	15.00
SD	0.05	06.73	01.77	13.72	13.36
Min.	1.58	47.00	17.57	08.00	12.00
Max.	1.75	70.00	24.61	60.00	60.00

Min. - minimum; Max. - maximum; SD – standard deviation; BMI – body mass index.

*One woman declared that she had never started professional orienteering training.

Questionnaires were collected from 49 WMOC medalists (21 women and 28 men), which represents 42.2% of all medalists from the WM35-WM75 (women and men aged 35–75) age classes (there were 116 medalists in the classes analyzed). Assuming a fraction level of 0.5 and a confidence level (α) of 0.95, the inference error from the obtained results was ± 11%, based on the analyzed group. We decided not to distribute surveys in the WM80 and older groups, as there may not have been enough participants in these age groups to fill the entire podium. No evidence was found that the return of surveys was not random. More detailed characteristics of the surveyed medalists are shown in Table 1.

### Methods

The research used a diagnostic survey method using a questionnaire developed by the first author and translated into English. The questionnaire author has extensive experience as an orienteering competitor (e.g., won three medals at the WMOC) and as a coach (e.g., coached the national junior team). The survey included questions regarding personal data, frequency and volume of training sessions, number of specific training sessions, and number of orienteering events. The questionnaire uses simple vocabulary, avoids abstract, ambiguous, slang, evaluative, and moralizing words, and excludes negations, institution names, and surnames. Furthermore, questionnaire items are specific and not too long, with each question covering only one issue. Three experts (orienteering coaches with over 30 years of experience) assessed the questionnaire and suggested minor changes to the possible answers to the partially closed questions (e.g., regarding the ranges of the number of training hours completed per week). Finally, we tested the questionnaire (a part that was used for this research) to determine its reliability. The result of the Cronbach’s Alpha test was 0.737, which is satisfactory [23].

The study was conducted in accordance with the Declaration of Helsinki and approved by the Senate Committee on Ethics of Scientific Research (approval code: 19/2021). Recruitment began on July 12, 2022, and ended on January 3, 2023. In the latter case, the survey was not anonymous. Almost all surveys were collected at the WMOC, with individual surveys sent by email within two weeks of its conclusion, and the medalists submitting the completed forms in paper form or electronically. Individual missing data were completed in surveys until the end of the year in which the championships took place. The questionnaire was distributed during the prize-giving ceremony (right after leaving the podium) and collected in a box that was displayed in the competition center over the following days. A few questionnaires were sent by email to the first author of this survey. Three people did not answer the question on training frequency during the competition period. The reason was probably that they considered the previous answer to the question on the frequency of training during the preparation period to be the same and did not repeat it. However, the missing answers were treated as actual missing data, and the remaining 46 answers were analyzed.

The Tanita MC 780MA S segmental multifrequency body composition analyzer assessed body weight, while the Seca 220 stadiometer (Secca GmbH & Co. KG, Hamburg, Germany) measured body height.

### Statistical analysis

Descriptive statistics characterized the groups of men and women, with all data analyzed using non-parametric tests since elite athletes do not fit the normal distribution typical of large populations, and the relatively small sample size. Moreover, some data did not have a normal distribution, which was confirmed using the Shapiro-Wilk test. Therefore, the Mann-Whitney U test with continuity correction assessed the significance of differences between men and women and examined differences between the two structural indicators, evaluated quantitative variables such as age classes, and the Kruskal-Wallis test with Dunn’s post hoc test assessed the magnitude of the influence of individual variables on the analyzed relationships between age, gender, and training variables.

For most statistical analyses, the subjects were grouped into four subgroups based on age and sex, including younger (WM35-WM55) and older (WM60-WM75) subgroups. As such, the subgroups included between nine and 16 people, which enabled statistical comparisons to be made using non-parametric tests. The division into two subgroups was also justified by biological reasons related to the aging process. The World Health Organization (WHO) considers the beginning of old age to be 60 [24], beyond which involutionary processes intensify significantly, which is certainly reflected in WMOC medalists. The level of Statistical analysis employed Statistica 13.3 PL software (StatSoft Polska, Kraków, Poland), with significance set at **p* *= 0.05.

### Methodological limitations

We excluded the 80-plus age classes because the number of participants in these classes is decreasing significantly, and their inclusion could have drastically affected the quality of the data obtained (the study would include people who did not represent the appropriate sports level, and winning a medal was only guaranteed by participation in the competition). Confounding variables, such as training history, injury status, and access to facilities, were not controlled in the study design.

## Results

### Medalist training frequency

**Fig 1:**
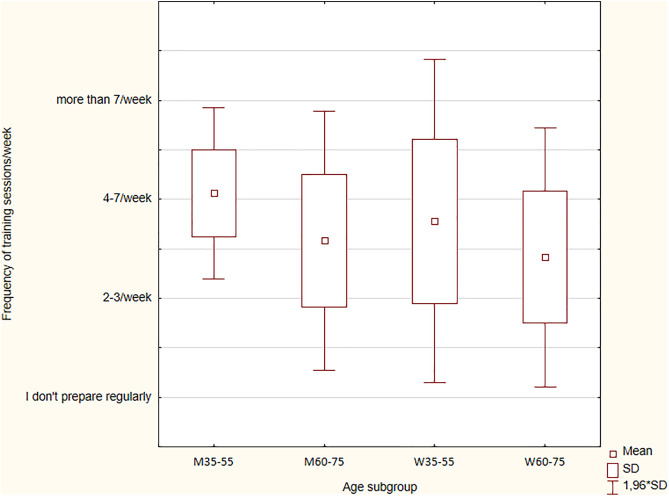
Differences in the number of training sessions across two age subgroups and sex.

One of the two parameters characterizing training loads (apart from intensity) is the volume of work performed during training. WMOC medalists were asked about the frequency of training and their approximate duration. These two parameters allowed us to estimate the training volume performed to properly prepare for the championship event (Fig 1).

Women usually trained two to seven times a week (81% of responses), with the proportions of those who trained two to three times a week and those who trained four to seven times a week evenly distributed (42.9%), while men mostly trained four to seven times a week (75%). When comparing the training frequency of men and women, some discrepancies were observed. A relatively higher percentage of men declared training sessions four to seven times per week (p = 0.015), and the opposite proportions were observed for two to three training sessions per week (p = 0.015). Among the WMOC medalists who trained more frequently, there were significantly more men than women after taking into account the difference in the number of respondents (comparing the proportions expressed as percentages, not absolute numbers). Four medalists (two women [9.9%] and two men [7.1%]) trained for the championships more than seven times a week, and all belonged to the WM35-WM55 subgroup.

When looking at training frequency based on age class, men and women from the WM35-WM55 subgroup trained most often, while women from the W60-W75 subgroup trained least. A lower training frequency was observed for both men and women in the WM60-WM75 subgroup. Significant differences in training frequency were found between the groups of younger and older men (Z = 2.080; p = 0.0037; η^2^ = 0.39). Such a relationship was not found in the group of women (Z = 0.812; p = 0.417). Additionally, the analysis of variance (ANOVA) of the Kruskal-Wallis test was performed, taking into account the division into four subgroups based on age and gender. No statistical significance for frequency of training was stated (H = 7.229; p = 0.065).

### Time devoted to training by medallists during the preparatory and the competitive phases

**Fig 2:**
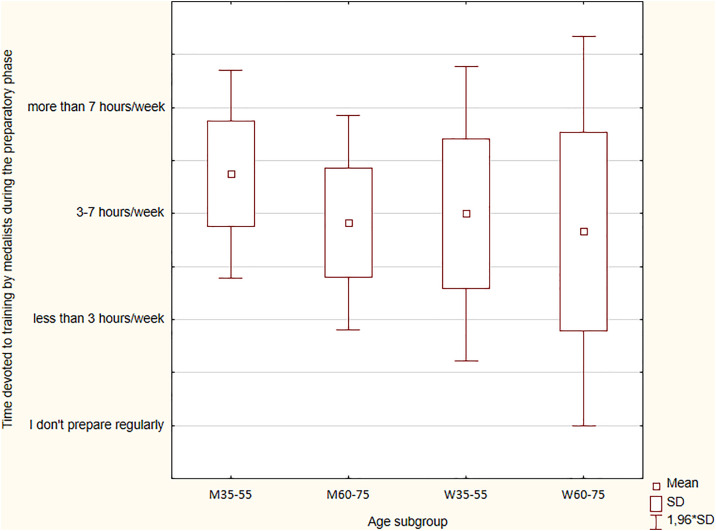
Time devoted to training by medalists during the preparatory phase.

**Fig 3:**
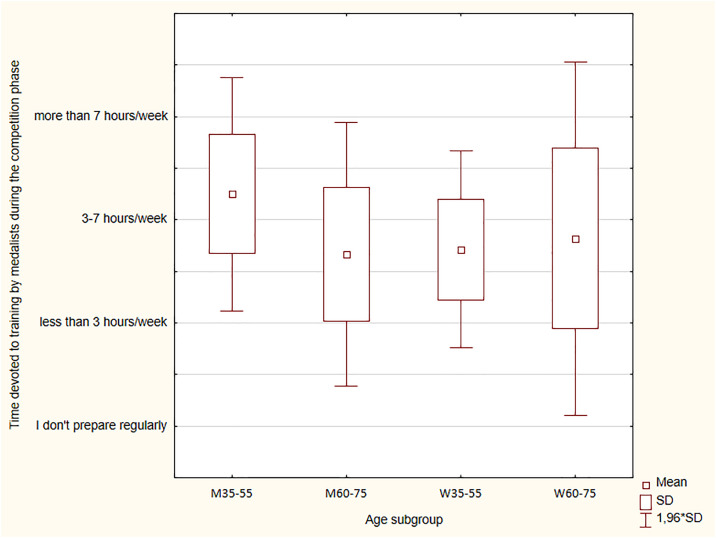
Time devoted to training by medalists during the competitive phase.

Orienteering participants were asked to specify the number of hours devoted to training per week, including the division into the preparatory and the competitive phases. The responses are presented in Figs 2 and 3.

During the preparatory phase, WMOC medalists usually trained from three to seven hours a week. Women declared less training volume than men in the preparatory phase (p = 0.045), which was determined by comparing the proportions of people declaring particular periods devoted to training each week. In the W35-W55 subgroup, 20% stated that they train less than three hours per week, while none of the M35-M55 subgroup trained so few hours (Fig 2). Proportions of women and men from the WM35-WM55 subgroup training three to seven hours per week were the same.

In the WM35-WM55 subgroup, almost 40% of men and more than 20% of women trained >7 hours/week, which was significantly higher than in the WM60-WM75 subgroup (p = 0.049) (Fig 2). There were no women in the W35-W55 subgroup who did not regularly prepare for the WMOC.

When comparing the time spent on training in the competitive phase to the preparatory phase, some differences were found. During the competitive phase, men and women devoted less time to training than during the preparatory phase (Figs 2 and 3). Particularly visible changes were observed in the men, where the proportions of people training fewer hours a week compared to those training more changed in the competitive phase, compared to the preparatory phase. More men and women were training less in the competitive phase compared to the preparatory phase. However, those who trained from three to seven hours per week still dominated in the WM35-WM55 subgroup and the WM60-WM75 subgroup. During the competitive phase, there were no significant differences in the declared training volume between men and women (p = 0.74). Additionally, the Kruskal-Wallis ANOVA showed no statistical significance for time devoted to training in the preparatory phase (H = 4.989; p = 0.173) and the competitive phase (H = 4.927; p = 0.177).

### Number of orienteering-specific training sessions in the six months preceding the WMOC

**Fig 4:**
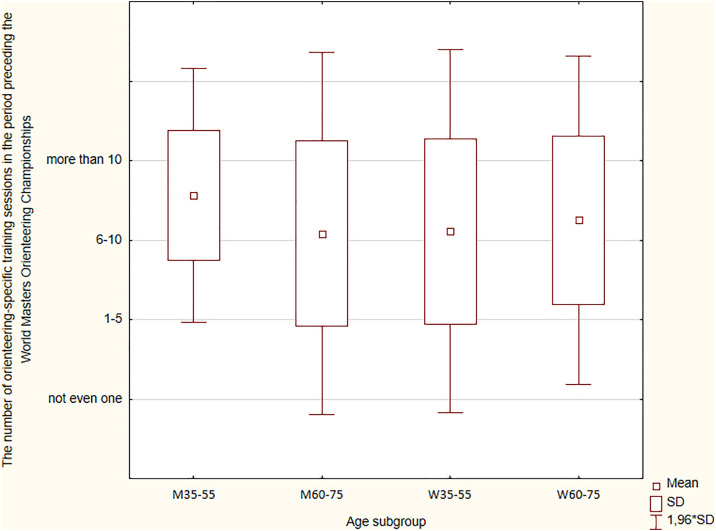
The number of orienteering-specific training sessions in the period preceding the WMOC.

From the point of view of sports practice, the number of specific training sessions aimed at improving technical and tactical skills important for orienteering task effectiveness (smooth, fast, and error-free navigation) is critical. Therefore, medalists were asked about the number of such training sessions in the six months preceding the start of the WMOC (Fig 4). It turned out that 62% of all medalists (54.5% of women and 67.9% of men) pointed to the largest range for specialized training proposed in the survey (> 10). The difference between men and women in this aspect of training was not significant (p = 0.333).

The most specific training sessions with a map were declared by men from the M35-M55 subgroup. In the WM65-WM75 subgroup, the number of specific training sessions was smaller compared to the WM35-WM55 subgroup. A comparison of younger and older WMOC medalists did not indicate significant differences between the women’s group. In the M60-M75 subgroup, no competitors declared that they had completed six to ten specialized training sessions, but one person did not undertake any specialized training at all (Fig 4).

According to the Kruskal-Wallis ANOVA, no statistical significance was found for the number of orienteering-specific training sessions (H = 1.723; p = 0.632). However, detailed analysis using Dunn’s test showed a significant difference between the W35-W55 subgroup and the M60-M75 (p = 0.009). Younger women from the W35-W55 subgroup used this type of training significantly more often than men from the M60-M75 subgroup.

### Number of orienteering competitions in the six months preceding the WMOC

**Fig 5:**
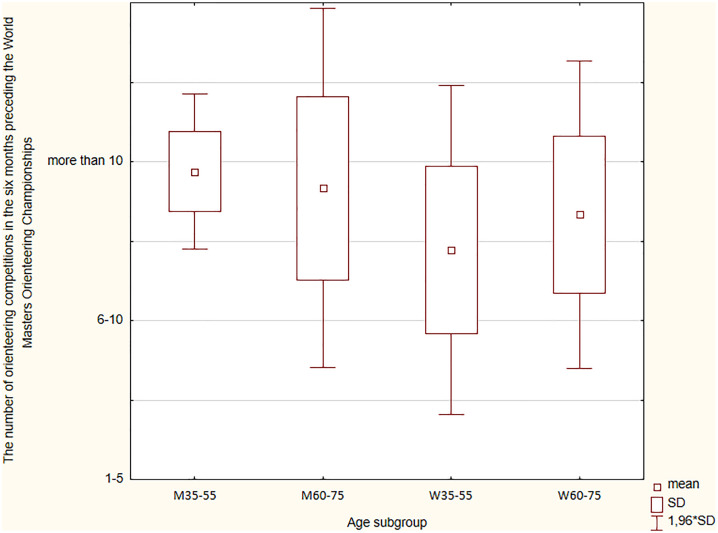
The number of orienteering competitions in the six months preceding the WMOC.

Women (54.5%) and men (92.9%) reported competing more than ten times in the six months preceding the WMOC (Fig 5), with this difference being statistically significant (p = 0.002). Female WMOC medalists competed less frequently in the six months before the championships than men. However, the comparison between the WM35-WM55 subgroup and the WM60-WM75 subgroup showed that women from the W60-W75 subgroup answered “more than 10 starts” more often than the W35-W55 subgroup, with no such phenomenon observed among men.

Each day of multiday or multistage orienteering competitions was counted separately. Using the Kruskal-Wallis ANOVA, statistical significance for the number of orienteering competitions in the six months preceding the WMOC was confirmed (H = 9.271; p = 0.026). Dunn’s analysis showed that the greatest differences were between men and women from the WM35-WM55 subgroup. Younger males were more likely to use this type of preparation method for the WMOC than younger female medalists.

## Discussion

The study aimed to capture the relationships between sports performance and workouts in different age groups of master foot orienteers. The analyses found that:

1. The average weekly training frequency of the WMOC medalists was four to seven times/week.
2. Training frequency was related to the age and sex of the medalists. Women aged 35–55 trained less or more frequently than four to seven times/week. Only the WM60–75 masters sometimes declare that they do not prepare regularly.
3. During the preparatory phase, the WMOC medalists usually trained three to seven hours per week. Women declared less training volume than men in the preparatory phase of the macrocycle (the annual training cycle).
4. In all subgroups, medalists used specialized training (running with a map) more than 10 times in the six months preceding the WMOC. Younger subgroups declared using this type of training routine at a higher frequency than older ones.
5. Men and women reported competing usually more than ten times in the six months preceding the WMOC. Female medalists (especially the group W35-55) competed less frequently in the six months before the championships than men in the same age subgroups.

According to Hébert-Losier et al. [25], peak performance age in orienteering ranged from 27 to 31 years, although much older competitors sometimes win medals at the elite world championships. However, after the age of 40, the chances of achieving significant results in orienteering decrease significantly, with the oldest medalist in the history of the world championships remaining 43-year-old Hakan Erikson. Bergström et al. [17] point out that the structure, culture, and nature of orienteering support a smooth transition from elite to recreational participation. Many former outstanding orienteers take part in competitions after finishing their professional careers, but in the master category. Many participants take part in orienteering competitions dedicated to masters, and these people have quite a high sports level when taking into account the involutionary changes related to aging. Bird et al. [26] stated that, before the age of 40, there was no substantial slowing in orienteering speed for males (0.5–4.2% per decade) but a moderate decline (4.7–10.0% per decade) for females. After the age of 45, the orienteering speed of males and females slowed by 13 ± 2% and 16 ± 4% per decade, respectively, until around the age of 69, after which the deterioration was accentuated.

In foot orienteering, age-related speed decline is greater than in other endurance sports [27,28]. In our opinion, the reasons may be twofold. The first is the strength nature of the effort, since off-road running requires a greater involvement of running strength than running on a treadmill or on a road. The second reason may be the fact that for many of those who practice orienteering, it is also a form of recreation, and they do not treat it as professionally as master athletes who compete in triathlon, running, swimming, or road cycling. According to Ganse et al. [29], the annual percentage rate of decline in track and field did not differ significantly between cross-sectional and longitudinal data, or between sexes in most disciplines. Performance declines after age 70 were 1.7 times (men) and 1.4 times (women) as steep as before [29]. De Veaux et al. [27] showed that men generally decline more slowly than women in athletics and swimming, while performance declines more rapidly for endurance events. In addition, athletes who participate more frequently decline more slowly than others, and master runners decline at rates roughly equivalent to world record holders. Different results regarding the rate of aging of different sexes and the impact on sports performance are presented by Gava et al. [30]. According to their research, the rate of deterioration in results achieved by men and women in athletics is similar. In foot orienteering, Nazário and Correia [31] observed substantial differences between men’s and women’s speed on the course, and these differences were more visible than in other endurance sports.

The current study did not compare the speeds achieved by women and men in the master category with each other. However, based on the results from the 2024 WMOC [32], the female from the W35 class speed was 135% of the speed of a man from the same age class on the classic distance. In the W75 class, the winner’s speed was 138% of the time of the male winner from the same M75 class. Furthermore, the speeds achieved were similar between men and women in the forest distance competitions among the WM35-WM55 subgroup and the WM60-WM75 subgroup. As such, it would be interesting to conduct trend analyses not only for the extreme classes of masters competitors, but to examine the trend for all masters classes in relation to the speeds achieved in orienteering races of various types and over various distances (sprint, middle, and classic).

Whether a reduction in training load accompanies a reduction in starting speed remains intriguing. Some authors state that a reduction in the volume of endurance work is one of the sources of decreasing maximal oxygen consumption (VO2max) among older athletes [33,34]. The analysis of training frequency and time devoted to training in the preparatory and the competitive phases showed that the number of training sessions and their duration decreased with age. Moreover, a clear difference emerged when comparing the training volumes of master orienteers with those of younger elite athletes, who reported engaging in 480 ± 65 training sessions per year during the season in which they won a world championship [35]. In contrast, even the youngest master athletes typically completed no more than 208–364 sessions annually, a range encompassing 87.7% of all male participants in the M35–55 subgroup. Among older masters, training frequency generally ranged from four to seven sessions per week, while over half of the women in this age group trained less frequently or irregularly. This reduced training volume likely played a key role in the observed decline in VO2max.

Men who won medals at the WMOC trained most often, four to seven times a week, and women trained two to seven times a week. Comparing this training frequency to elite world champions (women and men), it seems much less. It is clear that the WMOC medalists, classes from WM35-WM75, trained less than the elite athletes. When we compare the training frequency of orienteers from selected classes (e.g., the W35-W55 subgroup), several women declared training more than seven times a week. However, those in the W60-W75 subgroup no longer trained as often, indicating that training frequency decreases with age. Based on the analysis of training frequency, it can be concluded that master athletes practicing orienteering train less frequently than younger competitors (under 35 years of age), which is similar to observations from other endurance sports [36,37].

Another training load dimension analyzed was training volume, expressed as time devoted during the preparatory and competitive phases. Burtscher et al. [6] showed that the decline in VO2max with age was largely due to the reduction in training volume, which confirms previous observations made by Rogers et al. [33] and highlights why it is so important to maintain this component of training at the highest possible level. During the preparatory phase, the WMOC medalists usually trained for three to seven hours per week. The average training volume in the competitive phase was similar, but fewer people were training at this frequency compared to the preparatory phase. However, some people in the WM35-WM55 subgroup trained more than seven hours per week (40% of men and 20% of women). For comparison, elite orienteering world champions train an average of 15 hours per week in the preparatory phase and 11 hours per week in the competitive phase [35].

There is evidence [38] that an equally effective way to increase the effectiveness of master’s training is to maintain training intensity. Using such training in the master’s group maintains previous adjustments and positively affects anaerobic threshold and short-distance performance [36]. Celestino et al. [39], based on interviews with the world’s best orienteering competitors, determined that a critical aspect of striving for excellence in this sport is frequent contact with various types of terrain. In their discourse, they noted the importance of preferential contact with diverse mapping styles, terrains, and forms of reliefwhat makes an excellence in this sport. Over 62% of the WMOC medallists had more than ten specialized map training sessions in the six months preceding the competition, dominated by the M35-M55 subgroup (75%). However, some of the WMOC medalists did not undertake such training, and their only contact with the map took place during the competition. This is astonishing and seems almost unbelievable, yet possible. Such people were found in both female subgroups and the M60-M75 male subgroup. For comparison, elite world champions trained with the map for an average of 4.3 hours per week [35] and, according to Shirakov and Belomazheva-Dymitrova [40], 56% of the world-class foot orienteers participate in “between three and seven competitions” on a monthly basis, and 29.2% of the surviving competitors train “between three and seven times” on an average monthly basis. Based on this data, we can safely say that there is a substantial difference in the time devoted to specialized training with a map by most medalists and professional foot orienteers (elite world champions).

Another important factor in orienteering routine is off-road running, which significantly increases or maintains the strength of the muscles involved. Strength training can produce excellent results because master athletes may be able to maintain and increase strength in situations where strength training has not been previously engaged in [41]. Aging is characterized by a steady loss of muscle strength, which gets steeper from 70 years [42]. As such, maintaining or even increasing the amount of training aimed at developing strength is crucial late in the orienteering career.

The last of the analysed WMOC preparation elements was the number of starts in orienteering competitions in the six months before the championships. More than half of the women and almost all of the men competed more than ten times in the six months preceding the WMOC. Therefore, participation in competition was a way to achieve excellence for some master orienteers, especially since some did not use parallel-specific orienteering training during this period. Interviewed by Celestino et al. [39], the world’s leading competitors have repeatedly emphasized that participation in competitions over various terrains is one of the most effective ways to achieve mastery. According to Tønnessen et al. [35], world champions devote an average of 48 hours a year to participating in orienteering competitions, and 1.4 and 1.5 hours per week in the pre-start and start periods, respectively, which corresponds to approximately one or two starts and types of events a week (depending on the distance).

It is worth considering what influence the results obtained had on the previous sports experience of the medalists on their current preparations for the WMOC. We know from the questionnaires that several were former world championship medalists in the elite category when they were still young. These people certainly trained hard for their success. Perhaps they transfer these habits to their current lifestyle (including a professional approach to participation in non-qualified sports). Among the respondents, there was also a large group of former competitors who did not achieve significant successes on the international stage, but they also had previously instilled and deeply rooted training habits. Therefore, the current portrayal of how master orienteers prepare for participation in the WMOC is determined by these previous experiences. The influence of previous training and map-reading skills on results in the masters category may be another direction for researchers to explore.

### Strengths and limitations

Certainly, a limitation of our research is the fact that it is based on the declarations of the surveyed athletes, and not directly on facts (e.g., training diaries). On the other hand, the subjects were adults with many years of training experience and had no interest in providing false information. Another limitation of the research may also be the lack of full information on respondents’ physiological indicators (e.g., VO2max and maximum heart rate), which would allow for a more complete characterization of the subjects. In the current state of knowledge, we only know that they won a medal at the WMOC, but we would like to know much more about them. The study covered most, but not all, classes of master’s orienteering. The oldest (over 80 years of age) were not taken into account because of their much smaller numbers. The weakness of the study is its cross-sectional nature. That is why further observations will be needed to confirm the trends and differences observed by the authors. The strengths of the research include the fact that this is the first study of this type conducted among people practicing sports at an international level in the masters category in foot orienteering and, we will risk saying, the only one in the world in which it was possible to collect data from almost 50 medalists of the world championships in the masters category in one sport.

## Conclusions

The WMOC medallists trained to the best of their abilities, which decreased with age, and used various means in their preparations. Participation in orienteering competitions was quite common. Significant differences were observed between the volume and specificity of training used by medalists from subgroups separated by age and sex.

This finding implies that older people who are going to begin their adventure with foot orienteering in older age should adjust the volume and frequency of training sessions to their age group and place greater emphasis on training intensity to maintain or develop their VO2max. More strength training is also recommended, especially for those who have never participated, because orienteering is a sport that requires strength endurance to move around in the terrain.

To expand the knowledge about how master-aged athletes prepare for competition, it would be worth continuing this type of research in the coming years. It would also be advisable to include direct measurements of performance and other morpho-functional parameters, as well as parameters characterizing psycho-motor skills and cognitive processes.

## Supporting information

S1 Appendix Survey questionnaire. (DOCX)
