# Supplementary material for: Training processes of World Masters Orienteering Championship medalists
Source: PLoS One. 2025 Sep 25;20(9):e0333126. doi: 10.1371/journal.pone.0333126 (PMC12463196; doi:10.1371/journal.pone.0333126)
Supplement: S1 Appendix — (DOCX) [file pone.0333126.s001.docx]

**Survey questionnaire**

The purpose of this survey is to determine who the medallists of the Masters World Orienteering Championships are and what their training process looks like. Completing the questionnaire is voluntary, but its completion will certainly help to characterize the figure of the master in the orienteering run. Therefore, I encourage you to take a few minutes to complete it. Please fill in the dotted lines. In the case of lack of knowledge in a given field, please select the answer "I have no idea". No personal data will be disclosed. Thank you in advance for your time and contribution to the development of knowledge.

Year of birth …………. or age category …………. gender …………….. (Men / Women)

Body height ……….. (cm) body weight ………… (kg)

1. At what age did you start taking part in orienteering competitions? ……………….. (years)

2. At what age did you start regular training? …………………….. (years)

⃝ I have never started regular orienteering training

3. What is your greatest orienteering achievement in life?

…………………………………………………………………………………………………………………………………………………………….…………………………………………………………………………………………………………………………………………………………….

4. How old were you when you ended your sports career? ……………. (age in years)

⃝ I have never been a competitive (professional) orienteer

⃝ I have not finished yet

**Preparations for participation in this year's World Masters Championship in orienteering**

5. How many competitions (events) did you take part in the six months preceding WMOC? (if you participated twice in the competition on one day, count two starts and not one, please)

⃝ not once

⃝ 1-5

⃝ 6-10

⃝ more than 10

6. Do you train regularly, and if YES, how many times per week? (orienteering races and other competitions also include in it)

⃝ I don’t prepare (train) regularly

⃝ 2-3

⃝ 4-7

⃝ more than 7

7. How many hours do you spend on average training a week? (hours/week)

| - out of foot orienteering season (in the preparatory period) | - in the competition season |
| --- | --- |
| ⃝ I don’t prepare (train) regularly | ⃝ I don’t prepare (train) regularly |
| ⃝ less than 3 hours/week | ⃝ less than 3 hours/week |
| ⃝ 3-7 hours/week | ⃝ 3-7 hours/week |
| ⃝ more than 7 hours/week | ⃝ more than 7 hours/week |

8. How many orienteering training sessions (with the map and/or with a compass) have you done in the six months preceding WMOC? (excluding participation in competitions/events!)

⃝ not even one

⃝ 1-5

⃝ 6-10

⃝ more than 10

That’s all, thank you
